# Leadership Training to Increase Need Satisfaction at Work: A Quasi-Experimental Mixed Method Study

**DOI:** 10.3389/fpsyg.2019.02175

**Published:** 2019-09-25

**Authors:** Susanne Tafvelin, Ulrica von Thiele Schwarz, Andreas Stenling

**Affiliations:** ^1^Department of Psychology, Umeå University, Umeå, Sweden; ^2^Medical Management Centre, Department of Learning, Informatics, Management and Ethics Karolinska Institutet, Solna, Sweden; ^3^School of Health, Care and Social Welfare, Mälardalen University, Västerås, Sweden

**Keywords:** basic psychological needs theory, leadership training, self-determination theory, need support, quasi-experimental design, focus group interviews

## Abstract

With a growing number of studies showing the applicability of the self-determination theory for various work and organizational outcomes, the next logical step is to investigate if and how employee need satisfaction at work can be purposefully increased through an intervention. The purpose of the present study was to test whether we could train managers’ display of autonomy, competence, and relatedness support toward employees and whether this resulted in improved employee need satisfaction, well-being, and job performance. Data were obtained from 37 managers (rated by *N* = 538 subordinates) assigned to either an experimental or control condition at three time points: before, during, and after the training. We also used focus group interviews to evaluate the experience of the training. The quantitative analyses showed no statistically significant improvement in managers’ display of needs support or employee need satisfaction. However, the qualitative data pointed toward important factors related to the implementation of need supportive leadership training that should be considered.

## Introduction

Satisfaction of the basic psychological needs of autonomy, competence, and relatedness have been found to be important for employee psychological growth and well-being at work (Baard et al., 2004; Niemiec et al., 2009; Hofer and Busch, 2011). According to self-determination theory (SDT), basic psychological needs are innate nutriments essential for fostering autonomous motivation (Deci and Ryan, 2000), and a recent meta-analysis suggested that need satisfaction is related not only to employee motivation and well-being but to important job attitudes and behaviors, including job satisfaction, commitment, performance, and turnover intentions (Van den Broeck et al., 2016).

Given the role that need satisfaction plays at work, interventions that can improve employees need satisfaction may be a way to improve employee well-being and performance (Deci et al., 2017). One well-known antecedent of need satisfaction at work is the support employees receive from their manager in terms of autonomy, competence, and relatedness (Gagné, 2014). The leadership training of managers’ need supportive behaviors therefore has the potential to enhance employee need satisfaction at work. Interventions to improve individuals’ need-supportive behaviors toward others have been successful in other settings (Su and Reeve, 2011), but the transfer of this knowledge into the leadership training literature is limited and deserves further research attention (Hardré and Reeve, 2009; Slemp et al., 2018).

The purpose of the present study is to evaluate a leadership training that aims to improve managers’ need-supportive behaviors toward employees and thereby increase employee need satisfaction. Using a quasi-experimental design including both quantitative and qualitative data, we seek to advance theory and research in two ways. First, we examine the outcomes of a leadership training that aims to increase the support of all three basic psychological needs. Previous studies have only examined the trainability of managers’ autonomy support (Deci et al., 1989; Hardré and Reeve, 2009), which is surprising given that SDT emphasizes the importance of all three needs (Deci and Ryan, 2000). In addition, a recent meta-analysis indicates that the three needs explain incremental variance over and above each other (Van den Broeck et al., 2016), suggesting that the needs are not interchangeable, making it important to incorporate support of all three needs in training. Second, we further the study of the implementation of leadership training based on SDT at work by examining what factors leaders found helpful and hindering in applying need-supportive behaviors after training. As previous interventions based on the SDT has mainly been conducted in other settings, training parents, physicians, or teachers, little is known of what should be considered when implementing need-supportive leadership training of managers at work.

### Basic Psychological Needs Theory

Basic psychological needs are defined within the SDT framework as “innate psychological nutriments that are essential for ongoing psychological growth, integrity, and well-being” (Deci and Ryan, 2000, p. 229). In the same way that physical needs (e.g., hunger, thirst) are important for individuals’ physical survival, need satisfaction is assumed to be critical for individuals’ psychological functioning (Van den Broeck et al., 2008). The need for autonomy involves the perception of acting with a sense of ownership of one’s behavior, an absence of pressure, and the ability to feel psychologically free. The need for competence refers to a sense of mastery over an environment, including feelings of effectively interacting with the environment and developing new skills. Finally, the need for relatedness represents a desire to feel connected with others, to be respected, to be understood, and to be securely attached to others (Deci and Ryan, 2000).

Given that the satisfaction of the three basic psychological needs is critical for psychological growth and well-being, need satisfaction is expected to elicit positive outcomes related to psychological functioning and well-being (Van den Broeck et al., 2008). In line with these theoretical assumptions, a recent meta-analysis demonstrated that employees’ perception of need satisfaction at work was related to favorable attitudes, well-being, job behaviors, and motivation (Van den Broeck et al., 2016). For example, in a study of Canadian nurses, the perceptions of need satisfaction at work positively predicted work engagement and negatively predicted burnout and turnover intentions 12 months later (Trépanier et al., 2015). The satisfaction of the needs of autonomy, competence, and relatedness was found to increase work role performance among workers in the Belgian service industry (Leroy et al., 2015). Further, Van den Broeck et al. (2010) found employee need satisfaction to be related to job satisfaction, organizational commitment, and vitality.

### Basic Psychological Needs Theory and Leadership

Although many social–environmental and individual factors may influence employees’ need satisfaction at work, one of the most influential factors is the support provided by their manager (Gagné and Deci, 2005). Within SDT, three types of managerial support—autonomy, competence, and relatedness—have been acknowledged as important for employees’ need satisfaction, well-being, and performance (Gagné, 2014). Autonomy support is displayed when managers acknowledge employees’ feelings and are able to understand their perspective, while providing opportunities for choice and meaningful rationales as to why tasks needs to be done (Deci and Ryan, 2008). Managers who support competence communicate clear expectations, provide understandable guidelines, instill a sense of competence, and provide employees relevant feedback (Deci and Ryan, 2008). Relatedness support involves showing genuine interest in employees and their well-being in terms of spending time, energy, and resources on them (Niemiec et al., 2009).

Empirical studies suggest that managers’ display of need support is an important predictor of employee need satisfaction at work (Gagné, 2014; Slemp et al., 2018). For example, in a study of workers in a Norwegian banking corporation, managers’ need support positively predicted employee need satisfaction and justice perceptions (Olafsen et al., 2015). Furthermore, managers’ autonomy support predicted need satisfaction which in turn was related to performance and psychological adjustment among banking employees in the United States (Baard et al., 2004). The importance of need support for follower outcomes, such as need satisfaction and well-being, has also been demonstrated in other relationships, such as need support from teachers to students (Kaplan and Madjar, 2017) and from parents to their children (Chirkov and Ryan, 2001).

### Training Leader Need Support

Although interventions to improve individuals need-supportive behaviors have been implemented among, for example, teachers (Reeve et al., 2004), medical school interns (Williams and Deci, 1996), and dentists (Halvari and Halvari, 2006), surprisingly few attempts have been made to develop managers’ need-supportive behaviors toward employees. We only identified two previous studies. First, Deci et al. (1989) trained managers in a Fortune 500 company in how to display autonomy support. The training consisted of 3 days of training over 2 months where 28 managers were trained in how to take employees’ perspectives, allow more group participation in decision-making, and provide informational feedback. The results revealed that managers became more autonomy supportive and employees reported greater job satisfaction and trust in top management after training compared to a control group of employees.

In a second study, Hardré and Reeve (2009) trained 12 managers in a Fortune 500 company how to increase their autonomy-supportive behaviors. The training comprised of two 1-h sessions 1 week apart. After the first training session, managers were handed a training booklet for individual study. Using an experimental design, post-tests 5 weeks after training revealed that the managers in the experimental group displayed significantly more autonomy-supportive behaviors compared to managers in the control group. Also, their employees showed increased autonomous motivation and engagement.

Although these findings are promising, a number of issues deserve further research attention. First, no study has explored the trainability of managers’ competence and relatedness support (Cheon et al., 2016). Previous studies have focused on training managers’ autonomy support, which may reflect the fact that the need for autonomy has received most attention in SDT research. However, given recent meta-analytic findings that the three needs explain incremental variance over and above each other (Van den Broeck et al., 2016), it is relevant to also examine the trainability of competence and relatedness support. Second, previous research has only examined a narrow set of leadership training outcomes, including employee autonomous motivation, engagement, job satisfaction, and trust. No previous study has examined whether increases in managers’ need support after training could also increase employee well-being or performance, which would be expected both theoretically and given the findings from correlational studies (Deci et al., 2017). Finally, a meta-analysis on autonomy-supportive interventions in general found that a number of factors were important for successful implementation, including individual factors, such as (autonomy) causality orientation and lack of experience, and training design factors, such as brief sessions, skill-based training, and multiple types of media used to deliver the training (Su and Reeve, 2011). Given that these findings are mainly based on training parents, teachers, or health care professionals in autonomy support, it is of interest to study the implementation of need-supportive interventions directed toward managers to examine factors that may help or hinder the successful implementation of SDT-based leadership training.

### The Present Study

The aim of the present study is to evaluate a need-supportive leadership training program. We examine (1) manager and employee outcomes of need-supportive leadership training, and (2) hindering and facilitating factors when implementing need-supportive leadership training. The present study seeks to contribute to the leadership training literature in general and the understanding of the need-supportive leadership training in particular. Based on our review of basic psychological needs theory and previous research on autonomy-supportive interventions, we propose that the leadership training will improve participating managers’ need-supportive behaviors in terms of autonomy, competence, and relatedness support. In line with basic psychological needs theory (Deci and Ryan, 2000) and previous empirical studies (e.g., Van den Broeck et al., 2016), we also suggest that increased need support after training is positively related to employee well-being and performance in terms of need satisfaction, engagement, job performance, and well-being.

#### Hypothesis 1

Need-supportive leadership training will increase managers’ and employees’ perception of autonomy, competence, and relatedness support.

#### Hypothesis 2

Need-supportive leadership training will increase employee need satisfaction, job performance, and well-being.

Finally, we use focus group interviews to capture managers’ perceptions of hindering and facilitating factors to display need support after training. Given that few attempts have been made to train managers in need-supportive behaviors, this information may be of vital importance for any future studies seeking to implement similar interventions.

## Materials and Methods

### Participants and Procedure

#### Managers

This study was set in a midsized municipality in northern Sweden. The leadership training was conducted as a part of the municipality’s annual leadership development program, and the participants in the present study were relatively newly employed (i.e., within the last 1–2 years) managers enrolled in this program. Participation in the training program was mandatory, whereas participation in the research study was voluntary. Thirty-eight first-line managers employed in various sectors (e.g., childcare, culture, education, elderly care, leisure) were invited to participate in the present study (see Table 1 for descriptive statistics), and all agreed to participate. Of these, 21 managers were assigned to an experimental group and 17 were assigned to a wait list control group. One manager in the experimental group quit his job during the training program; he and his employees were therefore excluded from the analyses.

**TABLE 1:** Characteristics of participants at baseline and descriptive statistics of the study variables.

	T1 (Baseline)				T2				T3			
	Control		Experimental		Control		Experimental		Control		Experimental	
	*M*	*SD*	*M*	*SD*	*M*	*SD*	*M*	*SD*	*M*	*SD*	*M*	*SD*
**Managers**												
Age	45.06	7.25	42.50	9.21								
Tenure as manager	5.53	8.84	6.45	6.58								
Number of employees	24.29	11.88	24.2	10.31								
Autonomy support^∗^	4.19	0.50	3.85	0.36	4.17	0.49	3.75	0.45	4.08	0.51	3.86	0.30
Competence support	3.57	0.53	3.54	0.40	3.67	0.54	3.50	0.35	3.61	0.49	3.65	0.35
Relatedness support	4.31	0.55	4.18	0.43	4.15	0.50	4.04	0.34	4.23	0.49	4.05	0.36
**Employees**												
Age	44.59	11.37	43.99	11.90								
Tenure	9.61	8.74	9.45	8.33								
Years with manager^∗^	0.52	0.67	1.01	0.84								
Autonomy support	3.72	0.87	3.85	0.82	3.80	0.79	3.85	0.84	3.79	0.81	3.84	0.78
Competence support	3.50	0.88	3.64	0.83	3.50	0.91	3.65	0.88	3.54	0.90	3.62	0.89
Relatedness support	3.97	0.85	4.09	0.83	3.99	0.83	4.06	0.84	3.98	0.84	4.00	0.86
Autonomy^∗^	3.88	0.56	3.76	0.67	3.79	0.60	3.75	0.66	3.83	0.59	3.74	0.56
Competence	4.15	0.51	4.19	0.54	4.12	0.60	4.09	0.60	4.03	0.49	4.15	0.58
Relatedness	4.18	0.71	4.03	0.72	4.12	0.73	4.00	0.72	4.04	0.68	4.02	0.67
Job satisfaction	2.82	0.56	2.75	0.55	2.81	0.53	2.75	0.57	2.81	0.57	2.73	0.58
Vigor	4.50	1.00	4.33	1.09	4.39	1.13	4.19	1.20	4.39	1.07	4.19	1.18
Burnout	2.35	0.76	2.34	0.74	2.41	0.86	2.41	0.82	2.32	0.81	2.37	0.79
Work performance	7.77	1.35	7.68	1.41	7.52	1.48	7.47	1.75	7.53	1.57	7.58	1.54

*^∗^Statistically significant difference between the control and experimental group at T1 (*p* < 0.05).*

We were unable to randomly assign managers to conditions because the managers in the experimental group were those commencing the leadership development program in the fall of 2015, whereas the managers in the control group were to commence the program in the spring of 2016. Due to these circumstances, we employed what Shadish et al. (2002) referred to as a non-equivalent comparison group design. This design lacks randomization but both pretest and (multiple) post-test data are gathered on the participants, and potential selection bias can be examined by looking at baseline differences between the groups and at the developmental trajectories of both groups over time (Shadish et al., 2002).

#### Employees

The participating managers’ employees were also invited to be part of the study. In total, 742 employees were invited to participate, and 538 employees accepted the invitation and responded to questionnaires at least once, rendering a total response rate of 72.5%. The mean age, tenure, and years with managers in the control and experimental groups are shown in Table 1. The distribution of males (control = 21.5%, experimental = 22%) and females (control = 78.5%, experimental = 78%) was similar between the groups. A larger percentage of the control group employees worked full time (79.3 vs. 62.4%). More employees in the control group had a university degree (74.4 vs. 51.1%) and fewer in the control group had a high school degree (23.3 vs. 45.8%), whereas a similar percentage had elementary school as the highest educational attainment (control = 2.3%, experimental = 3%). The experimental group had a noticeably higher response rate (83%) among the employees compared to the control group (61%). The number of respondents at each measurement point was T1 = 442, T2 = 386, and T3 = 332. Aside from the written information the employees received about the study, the managers were encouraged to communicate (at a workplace meeting, via e-mail, or via an internal website) to their employees why they choose to participate in the study and to provide a rationale for why the employees were invited.

Data were collected using a web-based survey administered to the managers and employees in the experimental and control group at three measurement points. The baseline survey was administered to both managers and employees approximately 4 weeks prior to the first workshop, the second was administered within a week after the second workshop (approximately 2 months after the baseline survey and half way through the training), and the third survey was administered approximately 2 months after the second survey (as a post-measurement).

### The Leadership Training Program

Regarding the content and design of the leadership training program, we relied on previous recommendations for SDT-based leadership interventions (Su and Reeve, 2011), leadership training (Avolio et al., 2009), and previous successful SDT-based interventions (e.g., Cheon et al., 2012, 2015). Specifically, these recommendations suggest that: (1) skill-based training with a focus on specific behaviors is more effective than theory-based training focused on cognitions and emotions for changing behaviors; (2) the training should focus on multiple elements of need-supportive behaviors; (3) training first-line managers is more effective than middle and higher level management; (4) less experienced managers show larger effects of training; and (5) supplemental activities between sessions can boost the effect of the intervention. All of these recommendations were incorporated as a part of the leadership training program to facilitate learning and increases in need-supportive behaviors.

The leadership training program spanned 5 months (October 2015–February 2016) and included two 2-day sessions 1 month apart and a third half-day session approximately 3 months after the second session. We designed the training program in collaboration with an experienced leadership and organizational consultant with a Ph.D. in psychology who also delivered the program with the assistance of two leadership developers employed by the municipality. These two leadership developers have an overarching responsibility for the municipality’s leadership development program and leadership policy. To ensure that content of the training would be relevant to our particular group of mangers, we interviewed six managers that would participate in the training. The interviews focused on expectations on the leadership training, situations they perceived as difficult in their leader role, and what they wanted to learn at training.

#### Part 1

The first session was a 2-day workshop. It started with a discussion about the participants’ expectations on the training program and the anticipated outcomes of participating (e.g., consequences of participation, transfer of new knowledge and skills, an increase in knowledge, experiences during the program, or merely participation without expectations of progress). Following these initial discussions, the participants prepared a brief presentation about their view on leadership, what others can expect from them as a leader, and their strengths and weaknesses as a leader. These initial activities were designed partly to help participants get to know each other and partly to create a shared mental model that included the participants’ expectations and views on leadership. A presentation given by the consultant followed, which was based on SDT and covered managers’ need-supportive motivating style toward employees, the basic psychological needs at work, the nature of work motivation according to the SDT, and related outcomes at work (e.g., performance and well-being). This activity included workplace examples of need-supportive and need-thwarting behaviors and empirical evidence of the benefits of a need-supportive motivating style. It also included small group discussions, where, based on their own experiences, participants tried to identify behaviors, contexts, people, and situations as need supportive or need thwarting. During the last part of the first session, the participants received feedback based on their employees’ ratings of the mangers’ need-supportive behaviors and the employees’ need satisfaction at work. The feedback on need support was intended to provide managers with an assessment of their own strengths and weaknesses, while the feedback on employee need satisfaction was intended to give an overview of what kind of support employees might be in need of. The managers also formulated an action plan with support from the consultant and leadership developers based on the feedback from the employees for the how and when they could practice need-supportive behaviors at work. As a part of the action plan, the managers presented and discussed the results from the employee feedback from the first and second session. Finally, the participants received a copy of a book on need-supportive leadership at work (Söderfjell, 2012) to read before the second session.

#### Part 2

The second session was also a 2-day workshop, which was conducted approximately 4 weeks after the first session. During the first hour, the participants sat in small groups of three to four and discussed their experiences of presenting the feedback results to their employees, as well as if and how the action plan was being followed. They also discussed questions with the other participants that had arisen from reading the book. Following this initial activity, a brief repetition was provided to reiterate the tenets of SDT (i.e., motivation, basic psychological needs, need support). The remainder of the second session focused on practicing need-supportive behaviors by performing skill-based training of specific behaviors. More specifically, the participants practiced using active listening, need-supportive communication of newly imposed rules and regulations, a need-supportive language when writing e-mails, role play involving being need-supportive in different situations that often occur at work, need-supportive feedback, and need-supportive feedforward. All of these activities were practiced using role play (except the e-mail exercise) in smaller groups (mostly in triads), and the participants discussed and provided feedback to each other. Toward the end of the second session, the participants had the opportunity to revise or update their action plan with the assistance of the consultants. The action plans thus included the activities they planned to perform until the next session.

#### Part 3

The third session was a half-day workshop conducted approximately 3 months after the second session. During the session, the participants once again received feedback based on their employees’ ratings of the managers’ need-supportive behaviors and their need satisfaction at work. This session also included oral and written evaluations of the leadership training program, as well as focus group interviews with the managers about their experiences from participating in the program.

### Measures

#### Manager Rated Outcomes

##### Need support

Need support was assessed with the 12-item Need Support at Work Scale (Tafvelin and Stenling, 2018). This instrument consists of three subscales that capture managers’ perceptions of their autonomy support (four items), competence support (four items), and relatedness support (four items) toward their employees. Responses were given on a five-point Likert scale, ranging from 1 (never or almost never) to five (always).

#### Employee Rated Outcomes

##### Need support

Need support was assessed with the 12-item Need Support at Work Scale (Tafvelin and Stenling, 2018). This instrument consists of three subscales that capture employees’ perceptions of their managers’ autonomy support (four items), competence support (four items), and relatedness support (four items). Responses were given on a five-point Likert scale, ranging from 1 (never/almost never) to five (always).

##### Need satisfaction

Need satisfaction was measured with the 13-item Need Satisfaction at Work Scale (Tafvelin and Stenling, 2018). This instrument consists of three subscales that are designed to measure the satisfaction of the need for autonomy (four items), the need for competence (four items), and the need for relatedness (five items) in work contexts. Responses were given on a five-point Likert scale, ranging from 1 (completely disagree) to 5 (completely agree).

##### Job satisfaction

Job satisfaction (e.g., “How pleased are you with your work prospects?”) was measured with a four-item scale from COPSOQ II (Pejtersen et al., 2010). Responses were given on a five-point Likert scale from 1 (to a very small extent) to 5 (to a very large extent).

##### Vigor

We assessed vigor (e.g., “At my work, I feel like I am bursting with energy”) with a three-item scale from the short version of the Utrecht Work Engagement Scale (Schaufeli et al., 2006). Responses were given on a seven-point Likert scale from 0 (never) to 6 (always or every day).

##### Exhaustion

Exhaustion (e.g., “Do you feel worn out at the end of the work day?”), which is considered the core aspect of burnout, was measured with the seven-item work-related scale from the Copenhagen Burnout Inventory (Kristensen et al., 2005). Responses were given on a five-point Likert scale, ranging from 1 (never or almost never or to a very low degree) to 5 (always or to a very high degree).

##### Work performance

We used a one-item question from the World Health Organization Health and Work Performance Questionnaire (Kessler et al., 2004) to assess self-rated work performance. Respondents were asked to rate their overall work performance during the past 4 weeks on a 0–10 self-anchoring scale in which 0 is defined as the “worst possible work performance” a person could have on this job and 10 is defined as “top work performance” on this job.

Measurement reliability (coefficient omega [ω]; McDonald, 1999) of all measures are displayed in Table 2. Coefficient omega has been shown to overcome deficiencies of the most common measure, coefficient alpha, and is therefore deemed as a practical alternative to alpha in estimating measurement reliability of the total score (see, e.g., Revelle and Zinbarg, 2009; Dunn et al., 2014; McNeish, 2018).

**TABLE 2:** Omega coefficients.

	**T1**	**T2**	**T3**
**Managers**			
Autonomy support	0.63	0.79	0.79
Competence support	0.80	0.76	0.78
Relatedness support	0.74	0.65	0.71
**Employees**			
Autonomy support	0.88	0.87	0.86
Competence support	0.88	0.90	0.90
Relatedness support	0.92	0.91	0.92
Autonomy	0.77	0.77	0.77
Competence	0.74	0.80	0.79
Relatedness	0.91	0.92	0.92
Job satisfaction	0.79	0.80	0.83
Vigor	0.89	0.92	0.90
Burnout	0.89	0.90	0.91

### Data Analyses

Welsch’s *t*-tests were used to examine selection bias at baseline using JASP version 0.8.4.0 (JASP Team, 2017). Mplus version 8.0 (Muthén and Muthén, 1998-2017) was used to estimate unconditional, conditional, and multigroup linear latent growth curve analysis (LGCA) to examine the effects of the intervention (see Bollen and Curran, 2006 for an overview of LGCA). The unconditional LGCA was used to assess the average starting point at baseline (i.e., intercept mean), variation around the starting point (i.e., intercept variance), the average rate of change over time (i.e., slope mean), and the variation around that change (i.e., slope variance). In the conditional LGCA, a dichotomous variable (control vs. experimental group) was included as predictor of the intercept and slope factors. In the multigroup LGCA, the intercept and slope factors were simultaneously estimated in each group, and the slope means were compared using the Wald test of parameter equalities (Buse, 1982). For the employee data, we used LGCA combined with an adjustment of the standard errors and goodness-of-fit model testing for clustering (i.e., the TYPE = COMPLEX option in Mplus; Muthén and Satorra, 1995). The robust full information maximum likelihood (Enders, 2010) estimator was used to account for non-normality and missing data. The model fit of the LGCA was evaluated using conventional model fit indices, such as the chi-square test, the comparative fit index (CFA), the Tucker-Lewis index (TLI), the root mean square error of approximation (RMSEA), and the standardized root mean square residual (SRMR). Traditional cut-off criteria with CFI and TLI values around 0.90 and SRMR and RMSEA values around 0.08 were used to indicate acceptable fit (Marsh, 2007).

### The Focus Group Interviews

The focus group interviews were conducted during the last day of the leadership training. The 21 participating managers were divided into four groups, with four to six managers in each group. A semi-structured focus group guide was used to elicit managers’ perceptions of hindering and facilitating factors, both at an individual and organizational level, and to display need support after training and lasted for about an hour. The focus group interviews were conducted by four master’s psychology students. They recorded, transcribed, and later analyzed the interviews under supervision of the research team, using thematic analysis as described by Braun and Clarke (2006). The thematic analysis followed the six phases of familiarizing ourselves with the data, generating initial codes, searching for themes, reviewing themes, defining and naming themes, and producing the final report.

## Results

### Descriptive Statistics and Baseline Differences Between the Control and Experimental Group

Descriptive statistics and baseline differences between the employees and managers in the control group and experimental group are shown in Table 1. The managers in the control group self-reported providing slightly higher levels of autonomy support compared to the experimental group managers (*M* = 4.19 vs. *M* = 3.85, Cohen’s *d* = 0.76) at baseline. No other baseline comparisons were statistically significant. The number of years each employee had with the manager was slightly lower among the control group employees compared to the employees in the experimental group (*M* = 0.52 years vs. *M* = 1.01 years, Cohen’s *d* = −0.63). Employees in the control group also reported slightly higher levels of autonomy need satisfaction compared to the employees in the experimental group (*M* = 3.88 vs. *M* = 3.76, Cohen’s *d* = 0.19). No other baseline comparisons were statistically significant.

### Primary Outcomes: Changes in Need Support

The primary outcome variable for both managers and employees was the changes in ratings of need support (see Tables 1, 3; see Supplementary Appendix Table 1 for model fit of all the estimated LGCA). We did not observe any statistically significant changes over time (autonomy support = −0.02, *p* = 0.481; competence support = 0.04, *p* = 0.157; relatedness support = −0.05, *p* = 0.151) or differences between the managers in the experimental and control group. Similar results were found among the employees, and no statistically significant changes over time were observed in the experimental group (autonomy support = −0.01, *p* = 0.906; competence support = −0.01, *p* = 0.760; relatedness support = −0.05, *p* = 0.279) or control group (autonomy support = 0.04, *p* = 0.287; competence support = 0.02, *p* = 0.567; relatedness support = 0.01, *p* = 0.839). We also did not observe any statistically significant differences between the employees in the control and experimental group. As such, we did not find support for our first hypothesis that need-supportive leadership training would increase leaders’ and followers’ perception of autonomy, competence, and relatedness support.

**TABLE 3:** Parameter estimates of the LGCA.

	**Intercept mean (S.E.)**	**Intercept variance (S.E.)**	**Slope mean (S.E.)**	**Slope variance (S.E.)**
**Managers**				
Autonomy support				
Unconditional linear	4.00^∗^(0.07)	0.17^∗^(0.06)	−0.02(0.03)	0.02 (0.02)
Competence support				
Unconditional linear^*a*^	3.55^∗^(0.07)	0.13^∗^(0.04)	0.04 (0.03)	*NA*
Relatedness support				
Unconditional linear^*a*^	4.20^∗^(0.08)	0.11^∗^(0.04)	−0.05(0.04)	*NA*
**Employees**				
Autonomy support				
Unconditional linear	3.81^∗^(0.07)	0.56^∗^(0.07)	0.01 (0.03)	0.06 (0.03)
Control	3.73^∗^(0.11)	0.63^∗^(0.12)	0.04 (0.03)	0.05 (0.04)
Experimental	3.85^∗^(0.08)	0.53^∗^(0.09)	−0.01(0.04)	0.06 (0.05)
Competence support				
Unconditional linear	3.59^∗^(0.07)	0.62^∗^(0.07)	0.00 (0.03)	0.09^∗^(0.03)
Control	3.49^∗^(0.12)	0.68^∗^(0.11)	0.02 (0.04)	0.08 (0.05)
Experimental	3.65^∗^(0.08)	0.57^∗^(0.07)	−0.01(0.04)	0.10^∗^(0.04)
Relatedness support				
Unconditional linear	4.05^∗^(0.06)	0.55^∗^(0.05)	−0.03(0.03)	0.06 (0.03)
Control	3.98^∗^(0.11)	0.58^∗^(0.09)	0.01 (0.03)	0.01 (0.03)
Experimental	4.10^∗^(0.07)	0.53^∗^(0.07)	−0.05(0.04)	0.09 (0.05)
Autonomy				
Unconditional linear	3.80^∗^(0.04)	0.29^∗^(0.04)	−0.01(0.01)	0.03 (0.02)
Control	3.87^∗^(0.05)	0.20^∗^(0.07)	−0.03(0.03)	0.01 (0.06)
Experimental	3.75^∗^(0.05)	0.34^∗^(0.05)	−0.01(0.02)	0.04 (0.02)
Competence				
Unconditional linear	4.17^∗^(0.04)	0.26^∗^(0.03)	−0.04^∗^(0.01)	0.03^∗^(0.01)
Control	4.15^∗^(0.05)	0.26^∗^(0.03)	−0.06^∗^(0.02)	0.05^∗^(0.01)
Experimental	4.18^∗^(0.06)	0.26^∗^(0.03)	−0.03(0.02)	0.02 (0.01)
Relatedness				
Unconditional linear	4.08^∗^(0.05)	0.45^∗^(0.06)	−0.03(0.02)	0.04^∗^(0.01)
Control	4.18^∗^(0.06)	0.43^∗^(0.09)	−0.07^∗^(0.03)	0.021 (0.02)
Experimental	4.02^∗^(0.07)	0.46^∗^(0.08)	−0.00(0.02)	0.04^∗^(0.02)
Job satisfaction				
Unconditional linear	2.78^∗^(0.04)	0.24^∗^(0.03)	−0.01(0.2)	0.02 (0.01)
Control	2.82^∗^(0.06)	0.27^∗^(0.04)	−0.001(0.03)	0.03 (0.02)
Experimental	2.76^∗^(0.05)	0.23^∗^(0.03)	−0.01(0.02)	0.02 (0.01)
Vigor				
Unconditional linear	4.39^∗^(0.08)	1.07^∗^(0.11)	−0.07^∗^(0.03)	0.16^∗^(0.05)
Control	4.50^∗^(0.11)	1.01^∗^(0.10)	−0.06(0.04)	0.18^∗^(0.03)
Experimental	4.32^∗^(0.10)	1.07^∗^(0.15)	−0.08^∗^(0.04)	0.14^∗^(0.07)
Burnout				
Unconditional linear	2.35^∗^(0.05)	0.55^∗^(0.05)	0.01 (0.02)	0.07^∗^(0.02)
Control	2.35^∗^(0.09)	0.59^∗^(0.06)	−0.00(0.02)	0.06^∗^(0.02)
Experimental	2.35^∗^(0.06)	0.51^∗^(0.07)	0.02 (0.02)	0.06^∗^(0.03)
Work performance				
Unconditional linear	7.69^∗^(0.08)	1.69^∗^(0.34)	−0.08(0.05)	0.41^∗^(0.16)
Control	7.76^∗^(0.14)	1.82^∗^(0.33)	−0.13(0.08)	0.74^∗^(0.15)
Experimental	7.66^∗^(0.09)	1.98^∗^(0.23)	−0.05(0.05)	0.42^∗^(0.05)

*^*a*^Inadmissible solution due to negative slope variance. Slope variance set to 0. ^∗^*p* < 0.05. NA, not applicable.*

### Secondary Outcomes

Next, we examined the effects of the intervention on several secondary and more distal outcomes (need satisfaction, job performance, and well-being) among the employees. We only report the statistically significant and meaningful results in the text. Model fit statistics are shown in Supplementary Appendix Table 1 and parameter estimates of all the LGCA are displayed in Table 3. Descriptive statistics of all secondary outcome variables are displayed in Table 1.

An unconditional LGCA showed that there was an overall decline in competence need satisfaction (−0.040, *p* = 0.003) over time. A follow-up multigroup LGCA indicated that the decline was larger in the control group (−0.06, *p* = 0.008) than the experimental group (−0.03, *p* = 0.103). The difference in slopes, however, was not statistically significant: χ^2^(1) = 0.91, *p* = 0.339.

A similar effect was found for relatedness need satisfaction, indicating a negative but non-significant overall decline (−0.03, *p* = 0.155) over time. Multigroup LGCA showed a negative and statistically significant slope in control group (−0.07, *p* = 0.015), but a non-significant slope in the experimental group (−0.00, *p* = 0.949). The difference in slopes was not statistically significant: χ^2^(1) = 3.46, *p* = 0.0630.

Vigor also showed an overall decline (−0.07, *p* = 0.007). The multigroup LGCA showed a negative but non-significant slope in control group (−0.06, *p* = 0.133) and a negative and statistically significant slope in experiment group (−0.08, *p* = 0.033). The difference in slopes was not statistically significant: χ^2^(1) = 0.12, *p* = 0.7271.

Although we found indications of a larger decline in competence and relatedness need satisfaction in the control group compared to the experimental group, we also observed a larger decline in vigor in the experimental group. Considered together, we did not find support of our second hypothesis that need-supportive leadership training would increase follower need satisfaction, job performance, and well-being.

### The Focus Group Interviews

A thematic analysis of focus group discussions about managers’ perceptions of hindering and facilitating factors to display need support after training resulted in eight themes: the benefits of using cases and role play, the use of feedback, the helpfulness of a mandatory training, the general benefits of meeting with other managers, the mixed role of theory, the lack of sufficient individualization, the lack of integration in the organization, and the interference of other tasks.

To facilitate learning and transfer, overall, the managers felt that using practical cases and role play was an important positive characteristic of the training. It grounded the training in their reality and helped make sense of the theoretical content. It also gave them an opportunity to reflect on their leadership and a chance to practice and refine their skills in smaller groups.

The use of 180-degree feedback was also overall appreciated: it facilitated the identification of areas in need of improvement, provided a framework that helped transfer theory to practice, and made both employees’ and managers’ tasks, responsibilities, and expectations more explicit. Nevertheless, the results were perceived as difficult to use based on the way the data were aggregated, the low response rate, and the perception that the feedback session did not provide sufficient support in the interpretation.

As for the format, the managers perceived it to be positive that the training was mandatory for all managers. They felt that this justified the time and energy they spent on the training. This was helpful in relation to their own manager, their employees, and themselves. They also appreciated that the fact that the organization had made the training mandatory, which conveyed that it was an important and prioritized activity.

In addition to these more training-specific comments, the participants also emphasized that meeting other managers was a benefit in itself. It helped create a sense of belongingness so that the group became a source of social support. The mix of experience in the group was also valuable, as it helped them reflect on their own leadership. Yet, it was also described that the support from others could have been utilized even more to promote transfer, for example, by creating forums where leadership could be discussed among peers.

The managers reported a mixed experience of SDT. The major benefit was that it challenged them to think in new ways about their leadership, showing that being a leader is something qualitatively different from being a manager. The theory also helped in the communication with employees. However, the concept of autonomy was difficult to grasp, such as in regards to volition. They struggled with reconciling that with the responsibilities that follow from being an employee and part of a group. They also felt that need-supportive leadership was codependent on how the employees act, or that it takes two to tango. Several managers described SDT-based leadership as demanding a lot of energy from the leader.

Three factors emerged as particularly important and as barriers to leadership development. First, there was a perceived lack of individualization. The managers described a predefined package that did not leave enough room for the consideration of individual differences, experiences, and needs. This was mentioned both by leaders with short- and long-term leadership experience. Similarly, they perceived that the training was not equally relevant for all types of work, and therefore, not sufficiently adapted to the different types of workplaces. Also, some felt that the theory did not provide a good philosophical fit for everyone; that is, that they did not feel comfortable with the theory on a personal level.

Second, several managers expressed that the training was insufficiently integrated into their organization. This included a lack of understanding from both higher management and their own managers, who seemed unaware of the content of the training and did not prioritize it or follow-up with it. The managers called for more explicit demands from the organization so that the expectations on their leadership development was more pronounced. With that lacking, the managers felt they received unclear or even conflicting messages about what type of leadership they were expected to perform, if any. Overall, there was a lack of external demands or requirements for change that hindered transfer of learning.

Third, the managers acknowledged that conflicting work tasks and a substantial work load interfered with their leadership development. Part of this was the inherent conflicting demands of being a manager and being responsible for the daily operations and administrations, as well as looking after employees; that is, leading, not only managing. Another part was that they felt that they spent most of their time putting out fires. Acute business always seemed to take precedence over providing need-supportive leadership.

## Discussion

The present study evaluated a leadership training program based on the basic psychological needs theory. The training spanned 4 months and included 5 days of training and was based on previous recommendations for need-supportive interventions. The quantitative results showed no statistically significant improvements in neither leaders self-rated need support nor employees ratings of need support, need satisfaction, well-being, or performance, contradicting our two hypotheses. Finally, we used focus group interviews with leaders who participated in the training to further understand our findings, which are discussed below.

### Theoretical Implications

Given the increasing number of studies showing that employee need satisfaction is related to a number of important outcomes, including well-being and performance (Gagné, 2014), learning if and how managers can be trained in displaying need-supportive behaviors is of major interest. Although need-supportive interventions have been successful in other contexts, such as health care and in schools, few attempts have been made to train managers in need support (Deci et al., 2017). Contrary to our hypotheses, our need-supportive training did not increase perceptions of managers need support, and thereby no increases were observed in employee need satisfaction, job performance or well-being. This is somewhat surprising, given that our training was based on the recommendations for SDT-based leadership interventions (Su and Reeve, 2011) in terms of skill-based training focusing on multiple elements of need support. One recommendation what we were unable to follow, was the set up with short sessions. We relied on two 2-day sessions and one half-day follow-up based on the format the organizations had for their leadership training programs. However, in line with the latest meta-analyses of autonomy-supportive interventions, where short interventions showed somewhat larger effects compared to longer ones (Su and Reeve, 2011), it may have been more efficient with a larger number of shorter sessions and this may be one explanation for the inefficiency of our training. Nevertheless, our study suggests that previous findings in other contexts cannot easily be translated into the work context without carefully considering in what ways leadership training in need-supportive behaviors differs from training in need-supportive behaviors in other contexts. The results from the focus group interviews reveal that there are a several factors to consider when implementing need-supportive leadership training at work, with some factors being more general in nature while others are more SDT specific.

First, although our findings are in line with previous studies on the benefits of role play and feedback in leadership training (Kelloway et al., 2000; Su and Reeve, 2011), our findings also suggest that need-supportive leadership training needs to be aligned with the organization. This includes a need for vertical, horizontal, and diagonal alignment (von Thiele Schwarz and Hasson, 2012). The results underline that the leadership behaviors being taught need to be congruent with the goals and objectives of the organization (vertical alignment). Next, antecedents and consequences need to elicit and reinforce the wanted behaviors (horizontal alignment). Finally, there is a need for interlocking between management levels so that the senior managers support, not contradict, the wanted behaviors (diagonal alignment). The importance of alignment with the rest of the organization has been emphasized in the frameworks of organizational interventions (von Thiele Schwarz et al., 2016), and we suggest that this knowledge needs to be incorporated in future need-supportive leadership training programs.

Second, some aspects of basic psychological needs theory (Deci and Ryan, 2000) may not easily be translated into the work context. For example, in our training, the leaders found it difficult to relate to volition, which is an aspect of autonomy support. This may be a pedagogical problem, suggesting that insufficient attention were given to how employee volition may look at work and that future need-supportive leadership trainings should elaborate much more on how employees may feel when they are their true selves at work. However, it may also be that volition is not as relevant in the work context compared to other context. We found no previous studies that examined the role of volition at work specifically, which may be explained by the fact that all aspects of autonomy are usually collapsed into an overall measure, but we suggest future research examine the role of volition among employees more closely to gain further understanding of this aspect at work. It could be the case that some aspects of need support are difficult to implement at work or that need supportive leadership training needs to be complemented with training also in other types of leadership skills. Future studies are needed to examine this.

Third, shedding light on an issue of interest to the leadership training literature in general, our findings suggest that mandatory leadership training may be preferable to a voluntary attendance policy. Although this may seem surprising, our findings resonate with the latest meta-analysis on leadership training, which found that a mandatory attendance policy increased organizational results (Lacerenza et al., 2017). Lacerenza et al. (2017) suggested that this may be due to that fact that a mandatory attendance policy increases attendance and the number of leaders who are actually exposed to training. Our findings shed additional light on this issue and suggest that there may be other mechanisms in play, as the leaders in our study emphasized that the mandatory attendance policy made it easier for them to prioritize training, both toward their employees in terms of explaining why they were absent from work and toward others in the organization who tried to put additional demands on them.

### Practical Implications

These result have a number of practical implications for leadership training and for the evaluation of such in general and in need-supportive leadership specifically. First, these results underline the requirement for the alignment of the intervention with the organization. Making the training mandatory could be one way to facilitate alignment: according to our respondents, it signals that the organization supports and prioritizes the intervention. A current framework for the design, implementation, and evaluation of organizational interventions offers further input into how alignment can be created (von Thiele Schwarz et al., 2016). Building on this, we suggest that (1) intervention objectives are linked to organizational objectives in the planning phase, (2) the intervention is designed in collaboration with the participating organization to ensure a good fit and support alignment, and (3) barriers to alignment are managed up front by adding supporting interventions aimed at removing those barriers.

Second, the call for a higher degree of individualization has practical implications. One obvious way to increase individualization would be voluntary participation, albeit that would mean missing the potentially positive impact of having the mandatory training mentioned above. Therefore, we suggest increasing the flexibility in the program by applying the training activities that are used in the program to support need satisfaction in the delivery of the program. Practically, this could include focusing more explicitly on the participants’ personal goals, needs, and self and using dialogue to help them settle any congruence’s between the offered content and their needs.

Third, these results indicate the importance of considering the timing of the delivery of the intervention. Our intervention coincided with an increase in operative demands on the managers that stemmed from an unprecedented influx of people who are refugees: during 2015, the municipality received about 1,000 people who are refugees, in contrast to the 150 who had arrived the year before, which was similar to the increase of refugees at the national level (Statistics Sweden, 2016). This exceeded and worsened the always-existing challenge of managing different managerial objectives. Practically, it may not be feasible to go ahead with this kind of training program during such times. Monitoring the organizational and societal context for extreme events and being mindful about its impact on the implementation and effect of an intervention is important, as is being prepared to postpone an intervention.

### Limitations and Future Research

The findings of the present study should be viewed in light of its limitations. First, our small sample of managers restricted the statistical power in some of the analyses. Hence, we encourage future research to replicate our findings in larger samples of managers. Second, due to restrictions based on the wishes of the organization we collaborated with, we were unable to randomly assign managers into either an intervention or a control group. As a consequence of our non-equivalent comparison group design, managers in the leadership training had 6 months longer tenure than managers in the control group and had worked longer with their employees. It is possible that this was an advantage for the managers in the intervention group, as they had spent more time with their employees before training. Third, the design of the leadership training may be another limitation. As previously mentioned, our training included two 2-day sessions and one half-day follow-up based on the format the organizations had for their leadership training programs. (1). Future trainings may benefit from a design with a larger number of shorter sessions.

Given the incorporation of SDT into work and organizational psychology, it is important that future studies continue to examine how interventions to improve manager needs support and employee need satisfaction can be implemented in organizations. Although need-supportive interventions have been successfully implemented in other settings (e.g., with exercise instructors, Ntoumanis et al., 2017), our study points toward the requirement to tailor these interventions to the work context. We encourage future studies to further elaborate on how these interventions may be designed and evaluated, and it may, for example, be beneficial to measure change closer to where it actually happens, perhaps by videotaping staff meetings for behavioral observations or by asking employees to fill out questionnaires right after staff meetings. It would also be of interest to compare need-supportive leadership training interventions to other types of interventions to increase employee need satisfaction including changing incentives and the physical or the psychosocial work environment, to examine which ones are the most cost-effective.

## Conclusion

Our findings raise questions about the possibility to train leaders in need-supportive leadership. In this, it adds to the current understanding of SDT at work, and particularly the role of employee volition. In addition, it also adds to the current understanding of the implementation of leadership interventions that may be applicable beyond need-supportive leadership training by pointing toward the importance of aligning the training to organizational objectives as well as individualization and contextualization.

## Data Availability Statement

The data that support the findings of this study are available from the corresponding author, ST, upon reasonable request.

## Ethics Statement

This study was carried out in accordance with the recommendations of the American Psychological Association ethical standards, with written informed consent from all subjects. All subjects gave written informed consent in accordance with the Declaration of Helsinki. The protocol was approved by the Regional Ethics Board at Umeå University.

## Author Contributions

ST designed the study and collected the data together with AS. AS did the data analyses, and wrote the sections “Materials and Methods” and “Results.” ST wrote the section “Introduction.” UT wrote the section “Discussion.”

## Conflict of Interest

The authors declare that the research was conducted in the absence of any commercial or financial relationships that could be construed as a potential conflict of interest.
