# Corrigendum: Leadership Training to Increase Need Satisfaction at Work: A Quasi-Experimental Mixed Method Study

**DOI:** 10.3389/fpsyg.2020.00937

**Published:** 2020-05-05

**Authors:** Susanne Tafvelin, Ulrica von Thiele Schwarz, Andreas Stenling

**Affiliations:** ^1^Department of Psychology, Umeå University, Umeå, Sweden; ^2^Medical Management Centre, Department of Learning, Informatics, Management and Ethics Karolinska Institutet, Solna, Sweden; ^3^School of Health, Care and Social Welfare, Mälardalen University, Västerås, Sweden

**Keywords:** basic psychological needs theory, leadership training, self-determination theory, need support, quasi-experimental design, focus group interviews

In the original article, there was a mistake in Table 1 as published. Autonomy support ratings for employees across the three time points were missing. There were also some minor formatting errors that have been corrected. The corrected Table 1 appears below.

**Table 1:** Characteristics of participants at baseline and descriptive statistics of the study variables.

	**T1 (Baseline)**				**T2**				**T3**			
	**Control**		**Experimental**		**Control**		**Experimental**		**Control**		**Experimental**	
	***M***	***SD***	***M***	***SD***	***M***	***SD***	***M***	***SD***	***M***	***SD***	***M***	***SD***
**Managers**												
Age	45.06	7.25	42.50	9.21								
Tenure as manager	5.53	8.84	6.45	6.58								
Number of employees	24.29	11.88	24.2	10.31								
Autonomy support*	4.19	0.50	3.85	0.36	4.17	0.49	3.75	0.45	4.08	0.51	3.86	0.30
Competence support	3.57	0.53	3.54	0.40	3.67	0.54	3.50	0.35	3.61	0.49	3.65	0.35
Relatedness support	4.31	0.55	4.18	0.43	4.15	0.50	4.04	0.34	4.23	0.49	4.05	0.36
**Employees**												
Age	44.59	11.37	43.99	11.90								
Tenure	9.61	8.74	9.45	8.33								
Years with manager*	0.52	0.67	1.01	0.84								
Autonomy support	3.72	0.87	3.85	0.82	3.80	0.79	3.85	0.84	3.79	0.81	3.84	0.78
Competence support	3.50	0.88	3.64	0.83	3.50	0.91	3.65	0.88	3.54	0.90	3.62	0.89
Relatedness support	3.97	0.85	4.09	0.83	3.99	0.83	4.06	0.84	3.98	0.84	4.00	0.86
Autonomy*	3.88	0.56	3.76	0.67	3.79	0.60	3.75	0.66	3.83	0.59	3.74	0.56
Competence	4.15	0.51	4.19	0.54	4.12	0.60	4.09	0.60	4.03	0.49	4.15	0.58
Relatedness	4.18	0.71	4.03	0.72	4.12	0.73	4.00	0.72	4.04	0.68	4.02	0.67
Job satisfaction	2.82	0.56	2.75	0.55	2.81	0.53	2.75	0.57	2.81	0.57	2.73	0.58
Vigor	4.50	1.00	4.33	1.09	4.39	1.13	4.19	1.20	4.39	1.07	4.19	1.18
Burnout	2.35	0.76	2.34	0.74	2.41	0.86	2.41	0.82	2.32	0.81	2.37	0.79
Work performance	7.77	1.35	7.68	1.41	7.52	1.48	7.47	1.75	7.53	1.57	7.58	1.54

* *Statistically significant difference between the control and experimental group at T1 (p <0.05)*.

The authors apologize for these errors and state that this does not change the scientific conclusions of the article in any way. The original article has been updated.

