# Supplementary material for: Leadership Training to Increase Need Satisfaction at Work: A Quasi-Experimental Mixed Method Study
Source: Front Psychol. 2019 Sep 25;10:2175. doi: 10.3389/fpsyg.2019.02175 (PMC6773884; doi:10.3389/fpsyg.2019.02175)
Supplement: Supplementary file 1 [file Table_1.DOCX]

Appendix Table 1

*Model Fit of the LGCA*

|  | *χ*^2^ | *df* | *p* | CFI | TLI | RMSEA [90% CI] | SRMR |
| --- | --- | --- | --- | --- | --- | --- | --- |
| **Managers** |  |  |  |  |  |  |  |
| Autonomy support | | | | | | | |
| Unconditional linear | 0.54 | 1 | 0.4647 | 1.00 | 1.11 | 0.00 [0.00, 0.39] | 0.02 |
| Conditional linear | 2.88 | 2 | 0.2376 | 0.95 | 0.86 | 0.11 [0.00, 0.36] | 0.12 |
| Competence support | | | | | | | |
| Unconditional linear^a^ | 1.38 | 1 | 0.7097 | 1.00 | 1.04 | 0.00 [0.00, 0.20] | 0.18 |
| Conditional linear^a^ | 3.56 | 4 | 0.4688 | 1.00 | 1.02 | 0.00 [0.00, 0.24] | 0.18 |
| Relatedness support | | | | | | | |
| Unconditional linear^a^ | 6.73 | 3 | 0.0809 | 0.89 | 0.89 | 0.18 [0.00, 0.37] | 0.38 |
| Conditional linear^a^ | 7.62 | 4 | 0.1067 | 0.88 | 0.82 | 0.16 [0.00, 0.32] | 0.30 |
| **Employees** |  |  |  |  |  |  |  |
| Autonomy support | | | | | | | |
| Unconditional linear | 0.17 | 1 | 0.6783 | 1.00 | 1.01 | 0.00 [0.00, 0.09] | 0.01 |
| Multigroup | 0.62 | 2 | 0.7345 | 1.00 | 1.01 | 0.00 [0.00, 0.09] | 0.01 |
| Competence Support | | | | | | | |
| Unconditional linear | 0.04 | 1 | 0.8399 | 1.00 | 1.01 | 0.00 [0.000, 0.067] | 0.00 |
| Multigroup | 0.50 | 2 | 0.7800 | 1.00 | 1.01 | 0.00 [0.00, 0.08] | 0.01 |
| Relatedness support | | | | | | | |
| Unconditional linear | 0.27 | 1 | 0.6062 | 1.00 | 1.01 | 0.00 [0.00, 0.09] | 0.01 |
| Multigroup | 0.31 | 2 | 0.8570 | 1.00 | 1.01 | 0.00 [0.00, 0.07] | 0.01 |
| Autonomy | | | | | | | |
| Unconditional linear | 0.04 | 1 | 0.1678 | 1.00 | 0.99 | 0.04 [0.000, 0.132] | 0.01 |
| Multigroup | 7.97 | 2 | 0.0186 | 0.97 | 0.91 | 0.11 [0.04, 0.19] | 0.02 |
| Competence | | | | | | | |
| Unconditional linear | 1.94 | 1 | 0.1638 | 1.00 | 1.00 | 0.04 [0.00, 0.13] | 0.02 |
| Multigroup | 5.85 | 3 | 0.1191 | 0.99 | 0.99 | 0.06 [0.00, 0.13] | 0.03 |
| Relatedness | | | | | | | |
| Unconditional linear | 0.34 | 1 | 0.5590 | 1.00 | 1.01 | 0.00 [0.00, 0.10] | 0.00 |
| Multigroup | 1.13 | 2 | 0.5184 | 1.00 | 1.01 | 0.00 [0.00, 0.11] | 0.01 |
| Job satisfaction | | | | | | | |
| Unconditional linear | 0.06 | 1 | 0.8143 | 1.00 | 1.01 | 0.00 [0.00, 0.07] | 0.00 |
| Multigroup | 0.24 | 2 | 0.8868 | 1.00 | 1.02 | 0.00 [0.00, 0.06] | 0.01 |
| Vigor | | | | | | | |
| Unconditional linear | 4.88 | 1 | 0.0272 | 0.99 | 0.98 | 0.09 [0.02, 0.17] | 0.01 |
| Multigroup | 2.39 | 4 | 0.6653 | 1.00 | 1.01 | 0.00 [0.00, 0.07] | 0.03 |
| Burnout | | | | | | | |
| Unconditional linear | 11.22 | 1 | 0.0008 | 0.98 | 0.93 | 0.14 [0.08, 0.22] | 0.02 |
| Multigroup | 11.53 | 3 | 0.0092 | 0.99 | 0.97 | 0.10 [0.05, 0.17] | 0.02 |
| Work performance | | | | | | | |
| Unconditional linear | 7.27 | 1 | 0.0070 | 0.90 | 0.70 | 0.11 [0.05, 0.20] | 0.02 |
| Multigroup | 7.11 | 6 | 0.3108 | 0.99 | 0.99 | 0.03 [0.00, 0.09] | 0.08 |

^a^Inadmissible solution due to negative slope variance. Slope variance set to 0. **p* < 0.05. NA = Not applicable
